# Model-Informed Precision Dosing: Conceptual Framework for Therapeutic Drug Monitoring Integrating Machine Learning and Artificial Intelligence Within Population Health Informatics

**DOI:** 10.3390/jpm16020076

**Published:** 2026-01-31

**Authors:** Jennifer Le, Hien N. Le, Giang Nguyen, Rebecca Kim, Sean N. Avedissian, Connie Vo, Ba Hai Le, Thanh Hai Nguyen, Dua Thi Nguyen, Dylan Huy Do, Brian Le, Austin-Phong Nguyen, Tu Tran, Chi Kien Phung, Duong Anh Minh Vu, Karandeep Singh, Amy M. Sitapati

**Affiliations:** 1 Skaggs School of Pharmacy and Pharmaceutical Sciences, University of California San Diego, La Jolla, CA 92093, USA; 2 Tylan Health, El Monte, CA 91732, USA; 3 College of Pharmacy, University of Nebraska Medical Center, Omaha, NE 68198, USA; 4 Department of Clinical Pharmacy, Hanoi University of Pharmacy, Hanoi 100000, Vietnam; 5 Saint Paul Hospital, Hanoi 100000, Vietnam; 6 Department of Sociology, Dartmouth College, Hanover, NH 03755, USA; 7 Huntington Hospital, Pasadena, CA 91105, USA; 8 Division of Biomedical Informatics, School of Medicine, University of California San Diego, La Jolla, CA 92093, USA

**Keywords:** pharmacokinetics, model-informed precision dosing, population health, artificial intelligence, machine learning, population pharmacokinetics, patient safety

## Abstract

**Background/Objective**: Traditional therapeutic drug monitoring is limited by manual interpretation and specific constraints like sampling at steady-state and requiring a minimum of two drug concentrations. The integration of model-informed precision dosing (MIPD) into population health informatics represents a promising approach to address drug safety and efficacy. This article explored the integration of MIPD within population health informatics and evaluated its potential to enhance precision dosing using artificial intelligence (AI), machine learning (ML), and electronic health records (EHRs). **Methods**: PubMed and Embase searches were conducted, and all relevant peer-reviewed studies in English published between 1958 and December 2024 were included if they pertained to MIPD and population-level health, with the use of AI/ML algorithms to predict individualized drug dosing requirements. Emphasis was placed on vulnerable populations such as critically-ill, geriatric, and pediatric groups. **Results**: MIPD with the Bayesian method represents a scalable innovation in precision medicine, with significant implications for population health informatics. By combining AI/ML with comprehensive electronic health records (EHRs), MIPD can offer real-time, precise dosing adjustments. This integration has the potential to improve patient safety, optimize therapeutic outcomes, and reduce healthcare costs, especially for vulnerable populations where evidence is limited. Successful implementation requires collaboration among clinicians, pharmacists, and health informatics professionals, alongside secure data management and interoperability solutions. **Conclusions**: Further research is needed to define, implement, and evaluate practical applications of AI/ML. This insight may help develop standards and identify drugs for MIPD to advance personalized medicine within population health informatics.

## 1. Introduction

Drug safety is an important quality-improvement goal continuously strived for by the Institute of Medicine [1]. Addressing drug safety from a population health perspective targets health conditions in specific social, economic, and cultural environments at local, state, and national levels. Patients can suffer from complications, such as severe adverse drug events, that result in harm or fatality at varying severity and rates. Recent evidence shows that ADEs remain a major problem, particularly in vulnerable populations. Up to 18% of hospitalizations among older adults are ADE-related, and one in six critically-ill children experiences at least one ADE [2,3]. However, a proportion of ADEs are preventable, with reported rates ranging from 28% to 80% [4]. The Institute of Medicine recommends comprehensive patient data systems by integrating complete patient safety data accessible to all healthcare organizations [1]. This information is crucial to deliver safe, individualized patient care. Healthcare professionals can capitalize on recent technological advancements to innovatively design and implement new approaches focused on improving drug safety. One such method is the integration of MIPD into population health informatics—the application of information technology and data analysis to manage and enhance the health outcomes of specific populations [5].

MIPD uses advanced quantitative methods with complex mathematical and statistical models and algorithms to optimize patient-specific drug dosing [6]. AI and ML are currently explored in many sectors of the human health industry, including diagnostics, prognostics, agriculture, devices, and drugs [7]. With the continuous technological evolution, the integration of AI/ML into MIPD can advance precision dosing of drugs to ensure patient safety, as advocated by the Institute of Medicine. As such, MIPD affords promise in potentially becoming the new standard for individualizing drug dosing in the most vulnerable patient populations, including pediatrics, geriatrics, and critically-ill patients [8,9,10,11]. In these populations, physiological changes and altered organ function due to sepsis, shock, or multiple organ failure can significantly affect drug metabolism and clearance, which can lead to unpredictable drug exposure, resulting in consistent and frequent dose adjustments. Particularly in premature infants, who are considered the most extreme pediatric population, rapid physiological development, including renal maturation, occurs within the first 4 weeks of life and amplifies this challenge [12].

By enabling timely access to patient information to formulate clinical decisions, population health informatics is a key feature of successfully implementing MIPD into the clinical setting. The right drug dose for a population, with implications for the individual patient, may dynamically change over time as new information becomes available. Documentation of the most current patient diseases (which can alter drug dosing), medications, adverse events, and outcomes allows for better understanding of longitudinal health trends in the individual patient and overall population. Integration of AI/ML can further promote intensive, real-time surveillance, assist patient assessments, and inform clinical decision support, while decreasing the burden on healthcare professionals to manually monitor patients. In addition, the storage of health data electronically underscores the utmost importance of data security to prevent unauthorized users from accessing protected patient information.

As effective healthcare delivery increasingly relies on precision medicine to individualize treatments using patient characteristics, the integration of AI/ML with digital health data is inevitable. This article provides a narrative literature review of a modernized approach to precision drug dosing using MIPD, inclusive of the principles, methodology, and stakeholders of this next-generation patient safety approach. The integration of AI/ML within population health informatics that connects MIPD and health information to the healthcare team and key stakeholders, inclusive of timely feedback loops, is presented.

## 2. Methods

PubMed and Embase searches were conducted using the following terms: pharmacokinetics, model-informed precision dosing, population health, AI, patient safety, and machine learning. All relevant, available peer-reviewed studies in English published between 1958 and December 2024 were included, particularly to provide foundational literature to contextualize modern developments. Experts in clinical pharmacology, population health, biomedical informatics, pharmacy, and physician specialists (when necessary) guided the content creation and writing of this article.

## 3. Historical Perspective

**Figure 1:**
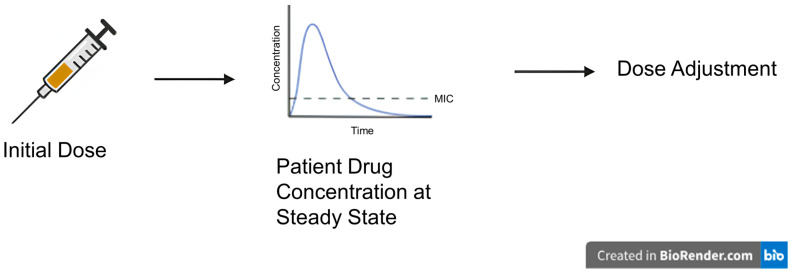
Traditional therapeutic drug monitoring based on patient drug concentration. Created with BioRender.com (https://www.biorender.com, institution version, accessed on 21 August 2025).

Therapeutic drug monitoring (TDM) has been used for many decades to individualize and optimize drug dosing by monitoring serum drug concentrations in patients [8,13]. TDM measures drug concentrations in various biological fluids and combines knowledge of pharmaceutics, pharmacokinetics (PK), and pharmacodynamics (PD) to interpret these concentrations within the context of relevant clinical parameters [14]. TDM requires blood draws to monitor serum drug concentrations to confirm exposure within the therapeutic range, with dose adjustment as needed to be defined (Figure 1). The exposure metrics used in TDM include maximum concentration (peak), minimum concentration (trough), and area-under-the-curve over 24 h (AUC_24_) that define optimal exposure by clinical trials with robust outcomes data [8,13].

TDM is often applied to drugs with narrow therapeutic windows to circumvent toxicity, high variability in PK, or potentially significant interactions with concomitant drugs. In addition to the clinical applications of TDM in various therapeutic areas, including anti-infectives, antiepileptics, chemotherapeutic agents, immunosuppressants, and anti-inflammatory drugs, TDM is invaluable in certain patient populations at high risk for adverse drug reactions, including pediatrics, geriatrics, and critically-ill patients [8,15,16,17]. The benefits of TDM are to achieve drug concentrations within the therapeutic window in a predictive manner, prevent supra- or subtherapeutic levels that may lead to toxicity or ineffectiveness, respectively, and ensure adequate clinical response to therapy. Current national and international guidelines recommend TDM, with expansion of the repertoire of drugs [16,17,18,19].

Despite the assistance of TDM, dosing errors and adverse drug effects have been reported for medications used in vulnerable patients, including those with impaired liver and kidney functions. Notably, approximately 30% of patients were underdosed and 26% overdosed with antibiotics in septic patients admitted to the intensive care unit [20]. In addition, many patients treated with tyrosine kinase inhibitors, a chemotherapeutic agent, experienced discontinuation or replacement treatment due to significant adverse effects, which led to drug resistance. MIPD, the next-generation TDM with Bayesian forecasting, has now been recommended in guidelines for vancomycin and beta-lactam antibiotics [17,18,21].

## 4. Model-Informed Precision Dosing

The concept of MIPD evolved from the efforts of Dr. Jelliffe RW, a cardiologist, and Dr. Sheiner LB, a pharmacologist [22,23]. In order to integrate MIPD into the clinical workflow, Dr. Jelliffe expressed a desire to expose computer-based tools and train future physicians and clinical pharmacists in MIPD [6]. With applications and benefits similar to traditional TDM, MIPD is the modernized approach to TDM used to predict personalized dosing with additional advantages in clinical practice [24]. First, blood sampling does not need to be performed at steady-state, allowing for early monitoring of drug concentrations and dosage adjustment that is advantageous for drugs with a long half-life and prudent in critically-ill patients who need a prompt response to therapy [25]. Second, a single drug concentration (usually the trough) rather than two drug concentrations (i.e., peak and trough) is adequate to evaluate drug exposure. This is helpful in neonates and children to minimize discomfort and blood volume from sampling [8].

### 4.1. Population-Based Pharmacokinetic Models

Clinical trials are conducted to create population-based PK models for specific drug-disease states [26]. These models contain a structural model that depicts the concentration-time profile of a typical patient, covariates (such as body weight, age, organ function markers, or concomitant medications) to enhance the final model, and adjustments for interindividual and intraindividual variabilities of the PK parameters [27,28]. In addition to simulation studies, the predictive performance of the population-based PK models can be improved using real-world data, accessible within population health informatics, where future drug exposure is predicted in the context of MIPD [25].

Multiple models established from these studies can be integrated with new data to build a more accurate model to represent the population. This is a particularly valuable approach in populations not well studied during clinical development (e.g., pediatrics, elderly, and pregnant patients) [26]. It is important to note that although MIPD tools are a valuable resource, healthcare providers will ultimately make the final clinical decision after consideration of the overall patient presentation, known as ‘provider in the loop’ [26].

Optimal management of drug safety in special populations requires an individualized approach that accounts for age-related physiological changes, comorbidities, current state of organ function, and hemodynamic stability for precise dosing [8,9,10,11,29,30]. When population-based PK models are available, healthcare providers must remain vigilant to ensure optimal therapeutic outcomes and, as equally important, drug safety in these vulnerable populations.

### 4.2. Workflow with Bayesian Forecasting

**Figure 2:**
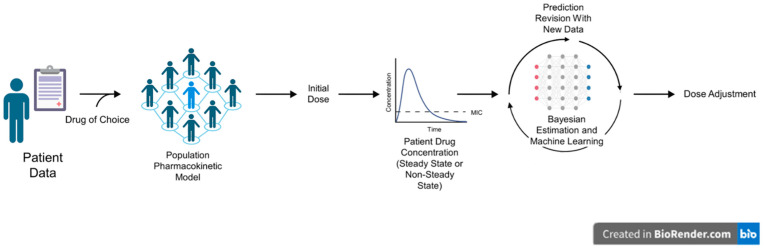
Model-informed precision dosing with Bayesian estimation integrated into ML. Created with BioRender.com (https://www.biorender.com, institution version, accessed on 21 August 2024).

The MIPD workflow is distinct from traditional TDM since MIPD utilizes a population-based PK model to predict an initial dosing regimen that maximizes the exposure targets. Selection of the right PK model is critical to enhanced accuracy; a neonatal PK model cannot be utilized in an older child whose age did not fall within the study population. MIPD accounts for multiple covariates, in addition to age, weight, and renal function, that are generally applied in dosing nomograms or tables used in TDM. Once measured drug concentrations become available, MIPD incorporates Bayesian forecasting and ML/AI to augment the precision of future drug dosing to achieve drug therapeutic and safety needs as quickly as possible to address the demands of acute care management (Figure 2). As such, the MIPD approach integrates a dosing framework tool to individualize treatment using both patient-specific data (i.e., serum drug concentrations) and population-based PK mathematical models with Bayesian forecasting [25].

Data standardization, from how it is collected to how it is stored, is crucial to prevent analytical errors and to ensure consistency within the MIPD tool. With advancing technology, there are more platforms for digital patient engagement and even home monitoring tools to deliver data to hospitals. The integration of these devices allows the collection of data at times that cannot be captured in healthcare settings. All these sampling points give more insight into a patient’s overall status and allow for more accurate predictions via MIPD commercially available software [26]. While multiple software have been developed for children and adults for this purpose, MIPD tools are not yet widely implemented for many drugs in clinical pharmacy due to cost prohibition [5,31,32].

Through integration of population-based PK models derived from clinical trials as the foundation for MIPD with the addition of Bayesian estimation and ML/AI within the EHR systems framework of population health informatics, precision drug dosing can deliver real-time with high accuracy, especially with rich digital data [33]. Furthermore, direct MIPD implementation applied via population health informatics can eliminate the need for time-consuming and error-prone manual data entry and enhance clinical efficiency [34].

### 4.3. Patient-Centered Care

MIPD is patient-centered, taking into consideration individual patient characteristics rather than relying on general one-size-fits-all dosing regimens. With this high individualization, MIPD is likely to satisfy patients’ health needs and desires. Furthermore, MIPD makes it possible for clinicians and patients to share decision-making fundamentals, enabling health professionals to support patients’ preferences, beliefs, knowledge, attitudes, emotions, and sociocultural context [1]. A layer of security is provided to the patients and their families when made aware of utilizing specific health-related information as an integral part of treatments.

A large portion of healthcare costs comes from medications that are needed for prevention and treatment. However, medication-induced health problems also significantly affect patients’ health quality and the economy [1]. Precision dose individualization optimizes patients’ treatments by limiting medication interactions and predicting the most effective dose. This should improve medication-induced issues, enhance patient health outcomes, and decrease costs [35]. In addition, the aging population, which is common in many countries, can also contribute to medication-induced health problems. As people age, organ function declines, leading to alterations in PK and PD. Thus, prior medications that have had a reliable effect in the past may not exhibit the same effect in the present. Hence, the implementation of MIPD in geriatric medicine is essential to minimize the harm caused by medications and potentially decrease the cost [10,35].

As MIPD is economical and beneficial to patients’ health, it should be provided to a broader population and made easily accessible to clinicians. However, obtaining a complete patient profile is time-consuming and is the main deterrent to using this approach to treat patients. Fortunately, the integration of clinical support systems and EHRs assists clinicians by giving easier access to patient information, which increases the accessibility to utilize dose individualization.

## 5. Machine Learning and Artificial Intelligence

Reliance on ML, the process of feeding data to a computer to train and generate a more efficient decision-making tool, is the primary mode with which AI is used to drive MIPD. During model development, AI can constantly refine its tool by predicting, testing, and evaluating its performance to improve its predictive capabilities. MIPD exploits this characteristic to achieve individualized drug dosing by integrating multiple parameters, including population-based drug PK, PD, and individual patient characteristics such as organ function, genetic information, and clinical conditions [36].

While ML has been widely used in many fields requiring intensive data collection, its role in pharmaceutical sciences and pharmacometrics modeling has not been emphasized. If ML is used effectively in MIPD, it will help to gather information from various necessary sources, such as public databases or disease registries, and make use of them by sorting out important data and clarifying their associations. In addition, it can also predict and learn from large and complex datasets. In this review, the integration of ML and AI in MIPD through recent research papers looking into ML use in pharmacometrics is presented [37].

Several ML algorithms have demonstrated utility in TDM and MIPD. Regularized regression techniques such as LASSO and elastic net facilitate covariate selection within complex clinical datasets. Tree-based ensemble learners, including random forests and gradient boosting machines, capture nonlinear patterns and high-order interactions relevant to drug exposure and toxicity [37]. Long-short term memory (LSTM) neural networks offer strong performance, incorporating longitudinal dosing and laboratory histories into exposure forecasting [38]. Rather than replacing mechanistic PK/PD models, these ML methods function as complementary tools enhancing exposure prediction, clinical risk stratification, and real-time decision support.

AI and ML also play a pivotal role within EHRs to approach individualized dosing. Predictive Model Markup Language was designed to represent various predictive models in a standardized language using extensible markup language, allowing the exchange of analytic models among different software platforms without a custom translation process [39]. This facilitates the ease of model use in healthcare settings. Moreover, precision medicine focusing on personalization of healthcare is accomplished through EHRs and order entry systems. Over the past decade, EHRs have evolved from basic adoption to advanced decision-support systems, incorporating shared data terminologies and facilitating clinical decision support, which enhances healthcare outcomes [40].

However, interpretability remains a critical prerequisite for clinical adoption of ML-based MIPD tools. Clinicians must discern the rationale behind model outputs and identify which patient factors drive dosing recommendations. Without transparency, even highly accurate models may be rejected in practice. Interpretability can be addressed using inherently transparent approaches such as generalized additive models, post-hoc explanation techniques including partial dependence plots and SHAP values, or hybrid frameworks embedding ML components within mechanistic PK/PD models [41,42]. These strategies support clinician trust and facilitate safe and accountable implementation.

Moreover, ML and AI in MIPD have been utilized in clinical settings with different models and applications. The chemotherapy patient population is prone to significant treatment toxicities, especially in renal and hepatic impaired patients, highlighting the importance of monitoring. A Multi-Layer Perceptron (MLP) model is an artificial network comprising an input layer of predictor variables and hidden connected layers of neurons that carry out calculations, analyzing relationships between variables to predict outcomes [43]. MLP demonstrates potential to optimize blood test strategies by minimizing unnecessary tests for low-risk patients while enhancing safety for high-risk individuals [43]. Additionally, in patients undergoing chemotherapy, an ML algorithm leveraging electronic health record data effectively predicts short-term mortality. While promising, future research should look at the generalizability and practical implementation of these tools in clinical settings [36].

Another AI and ML application is TRIPOD + AI, an updated version of TRIPOD (Transparent Reporting of Multivariable Prediction Models for Individual Prognosis or Diagnosis) guidelines. With the expansion of the 27-item checklist, TRIPOD + AI enhances transparency and accuracy in reporting studies along with the Abstracts checklist. It unifies guidelines for reporting studies on prediction algorithms, regardless of the use of either traditional regression methods or modern ML techniques. The emphasis of TRIPOD + AI is the comprehensive and precise reporting of studies to enable proper assessment, validation, and implementation of prediction models in practice [44].

## 6. Technological Advancement

Technological improvements in computing power, user interface, and sample/data collection exist in the current healthcare setting. An increase in computing power is utilized to process the MIPD data frameworks, guiding therapeutic decision-making for physicians and pharmacologists. Bayesian dosing in MIPD models provides an opportunity for AI/ML to further advance and improve algorithms. To achieve this, technological innovations must be revisited to update older Bayesian models as well. MIPD technology also needs to be user-friendly to be realistically implemented in modern clinical practice. The practicality of MIPD is extremely important for professionals to begin application to their daily routines, which can easily be achieved with modern technology. Finally, the quality of PK and biomarker samples used to create databases and information networks must be at their best. With proper and modern sample collection, the MIPD has a strong foundation of data to work with, producing optimized results for healthcare professionals [26].

Integration of MIPD tools into EHRs is ideal, as it will not require healthcare providers to manually input patient data. Systems must be affordable, easy to use, and timely and be capable of delivering high-performance computing to be widely implemented. Implementation studies are required to identify the most effective integration of MIPD strategies into real-world settings, such as providers’ clinical decision support and patient alerts. Further evaluations conducted will demonstrate the impact of these tools on clinical outcomes.

Despite these advances, important limitations still constrain ML applications in therapeutic drug monitoring (TDM). TDM datasets often suffer from small sample sizes, sparse and unbalanced sampling schedules, missing laboratory values, and censored concentrations, all of which increase susceptibility to overfitting and weaken external validity [37,45]. Site-specific treatment protocols, assay variability, and changes in clinical practice introduce dataset and concept drift that may undermine generalizability. Moreover, many published ML models lack rigorous external validation, calibration assessment, or prospective evaluation [37,46]. These shortcomings emphasize the importance of robust study design, harmonized reporting frameworks such as TRIPOD-AI, and multi-institutional validation before clinical deployment.

## 7. Population Health Informatics

### 7.1. Ecosystem/Infrastructure

A huge obstacle to ideal care is the fragmentation of patient health-related information. This can be attributed to disparate, non-integrated systems such as electronic health records, lab systems, patient home monitoring, and home health. Blockchain has been proposed as a method for secure data sharing between systems. First developed in 2008, it is a decentralized system with peer-to-peer technology; all users who join the network gain access to a copy of the chain. When new information is added, each node will validate it, and the new block will be added to the chain once a consensus is reached. Once added, the data cannot be modified. Such features make the network more secure. Implementation of this system could decrease the time needed to transfer and access data from other hospitals and allocate it back to healthcare professionals to focus on making patient care decisions. Health blockchain holds promise as a more cost-effective, efficient means to secure health information.

**Figure 3:**
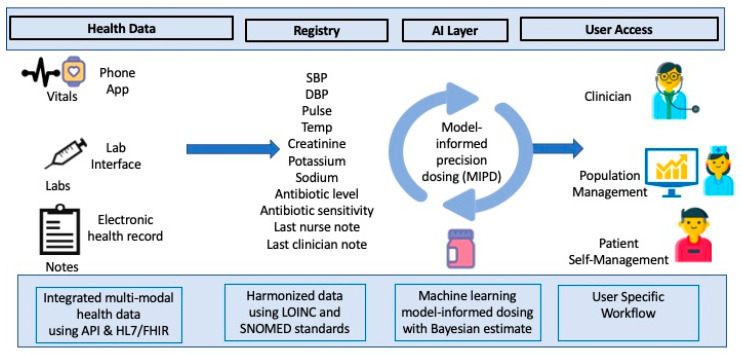
Population health informatics with the integration of AI into patients’ health records to support healthcare clinical decisions and patient accessibility.

Many other methods have been integrated to allow access to a complete patient profile, for example, the HL7 Fast Health Interoperability Resources (FHIR), a standard for exchanging electronic healthcare data [47]. Epic and Cerner, both major EHR software, integrate FHIR to some extent. These integrated systems allow for expedited lab result time, resulting in more timely and punctual interventions (Figure 3). Software must also be easy to implement and scalable to support widespread use.

Patient objective data from home monitoring (such as vitals) can now be monitored in real-time. This aggregate information can be seamlessly transferred into the EHR, providing day-to-day data and a broader overview of the patient’s condition.

The collection of data results and processing time also delays the care that can be provided for the patient. Delays in decision-making can result from samples sent to the hospital laboratory or an external laboratory, depending on the test. Contemporary point-of-care (POC) testing performed at the patient’s bedside accelerates the time from test to actionable result. This gain in efficiency can be further made via automatic integration of home-based POC device results to the EHR, additionally reducing documentation time for manual charting of results. ML algorithms are ripe to process this data and can further hasten precision dosing action.

### 7.2. Data Security

The sensitive information contained on EHRs requires security safeguards to be put in place to protect patient information per the Health Insurance Portability and Accountability Act (HIPAA). Access control prevents unauthorized parties from acquiring patient information through anti-malware and encryption [48]. Rigorous processes inclusive of dual authentication, encryption, and cloud-based systems further enhance security.

### 7.3. Workflow

Usability of applications is essential for efficient and effective care delivery. An intuitive design simplifies navigation and limits workarounds that clinicians feel compelled to use to bypass processes and complete their intended task [49]. Default views should be tailored to user roles and current activity in their view of the EHR to display relevant information and minimize overload. For example, pharmacists preparing for the third dose of an antibiotic infusion with narrow therapeutic margins should have the estimates of optimal dosing and interval displayed as they manage order management and lab review. In addition, ease of ordering labs and carrying out other necessary processes via the EHR can make a big impact on time efficiency; thus, optimizing such systems is important to lessen clinicians’ burden.

### 7.4. Key Stakeholders

Physicians and pharmacists have long acknowledged the presence of inter-individual variability in response to the same medications [50]. This variability comes from both intrinsic and extrinsic characteristics. Factors such as polygenic genes, liver, and renal function will change drug PK [50]. Other factors more easily manipulated, such as food intake, health status impacting albumin and renal function, and concomitant medications, also affect response. The various combinations of dynamic complex factors make it very difficult to determine the optimal dosage treatments for patients. With the development of modern biological inventions such as the Human Genome Project, it is now possible to determine genetic factors that cause differing PK, the most elusive piece in the variability puzzle [50]. Nonetheless, there are still many other challenges preventing clinicians from individualizing dosing. One of those challenges is due to a lack of robust dosing information from clinical trials. Pharmaceutical companies often conduct trials with a few dosing regimens. When there are recommendations for a certain group of patients (e.g., pediatrics), the regimen does not integrate other factors and assumes a uniform group response. Another challenge is the difficulty in monitoring patient outcomes due to time constraints, as well as the uncertainty of true outcomes and related biomarkers [5].

In addition to physicians and pharmacists, clinical pharmacologists are also the key figures whom MIPD can benefit via integration into population healthcare. Physicians and pharmacists can utilize MIPD to create more individualized healthcare plans for patients, and clinical pharmacologists can weigh different treatment and dosing options for optimized therapeutics. As the spearheaders of potential MIPD integration, physicians and pharmacologists can accelerate the clinical acceptance of MIPD Bayesian dosing within the healthcare community at large. The lack of pharmacometrics within many healthcare professional programs often causes indifference towards individualized dosing by many practices outside of pharmacy [35,51].

Therefore, MIPD, with its characteristics of learning and reasoning to predict optimal drug dosing, will become a great assistive tool for clinicians. Physicians have noticed the advantages of MIPD’s ability to predict the most appropriate patients’ initial dosage treatment, use patient drug levels to adjust the dose regimen to meet ideal targets, and aid in adapting new treatments [6]. MIPD is not only necessary to achieve the best outcomes in treating patients, but it also gives clinicians more time and flexibility to embrace novel therapeutics.

### 7.5. Process

Many logistical challenges must be addressed before MIPD can begin making its appearance in population-wide healthcare systems. One of these issues includes the creation of guidelines and regulatory practices for MIPD use. Regulatory agencies must clarify the status of Bayesian dosing systems and establish licensing to ensure proper use by healthcare professionals. The MIPD tools must also be consistently evaluated and updated to produce the best possible recommendations. From an economic standpoint, integration of MIPD requires sufficient funding and reimbursement frameworks that acknowledge the team roles to be implemented and reliably used population-wide. The clinical and economic benefits must be demonstrated through research publications to justify these investments [26]. Additionally, discussions must be made to determine the extent to which patient information can be shared amongst multiple healthcare providers as well as other forms of digital experience [1].

## 8. Conclusions

The successful implementation of MIPD requires reliance on interprofessional collaboration between pharmacists and clinicians and utilization of population health informatics. Systems that modernize through innovation in MIPD have the opportunity to reduce burden, increase efficiency, and improve patient safety and health outcomes.

## Data Availability

No new data were created or analyzed in this study. Data sharing is not applicable to this article.

## Abbreviations

The following abbreviations are used in this manuscript:

AI	Artificial Intelligence
AUC_24_	Area-Under-The-Curve Over 24 Hours
EHRs	Electronic Health Records
FHIR	Fast Health Interoperability Resources
HIPAA	Health Insurance Portability and Accountability Act
MIPD	Model-Informed Precision Dosing
ML	Machine Learning
MLP	Multi-Layer Perceptrons
PD	Pharmacodynamics
PK	Pharmacokinetics
POC	Point-of-Care
TDM	Therapeutic Drug Monitoring
TRIPOD	Transparent Reporting of Multivariable Prediction Models for Individual Prognosis or Diagnosis
